# Changes in conformational dynamics of basic side chains upon protein–DNA association

**DOI:** 10.1093/nar/gkw531

**Published:** 2016-06-10

**Authors:** Alexandre Esadze, Chuanying Chen, Levani Zandarashvili, Sourav Roy, B. Montgometry Pettitt, Junji Iwahara

**Affiliations:** Department of Biochemistry and Molecular Biology, Sealy Center for Structural Biology and Molecular Biophysics, University of Texas Medical Branch, Galveston, TX 77555-1068, USA

## Abstract

Basic side chains play major roles in recognition of nucleic acids by proteins. However, dynamic properties of these positively charged side chains are not well understood. In this work, we studied changes in conformational dynamics of basic side chains upon protein–DNA association for the zinc-finger protein Egr-1. By nuclear magnetic resonance (NMR) spectroscopy, we characterized the dynamics of all side-chain cationic groups in the free protein and in the complex with target DNA. Our NMR order parameters indicate that the arginine guanidino groups interacting with DNA bases are strongly immobilized, forming rigid interfaces. Despite the strong short-range electrostatic interactions, the majority of the basic side chains interacting with the DNA phosphates exhibited high mobility, forming dynamic interfaces. In particular, the lysine side-chain amino groups exhibited only small changes in the order parameters upon DNA-binding. We found a similar trend in the molecular dynamics (MD) simulations for the free Egr-1 and the Egr-1–DNA complex. Using the MD trajectories, we also analyzed side-chain conformational entropy. The interfacial arginine side chains exhibited substantial entropic loss upon binding to DNA, whereas the interfacial lysine side chains showed relatively small changes in conformational entropy. These data illustrate different dynamic characteristics of the interfacial arginine and lysine side chains.

## INTRODUCTION

DNA recognition by proteins is vital for gene expression, DNA replication and repair. Three-dimensional (3D) structures of protein–DNA complexes show that basic side chains play important roles through electrostatic interactions with DNA phosphates as well as hydrogen-bonding with DNA bases (1–3). Thermodynamic studies also indicate the importance of interfacial basic side chains: they form ion pairs with DNA phosphate groups and cause release of condensed counterions from DNA, which is a driving force for many protein–DNA association processes (4–6).

Despite the importance of the basic side chains, their dynamic properties have not been well studied by experimental means. Although some studies by nuclear magnetic resonance (NMR) spectroscopy show significant roles of conformational entropy in macromolecular recognition and association (7–9), such investigations typically probe the dynamics of backbone NH or side-chain CH_3_ groups only. For side chains that form hydrogen bonds and/or ion pairs, the dynamic properties and their entropic roles remain largely unknown. This represents a bottleneck to thoroughly understand molecular recognition of nucleic acids by proteins, where a large number of intermolecular hydrogen bonds and electrostatic interactions are involved.

From this perspective, we conduct a comparative study on the conformational dynamics of arginine (Arg) and lysine (Lys) side chains of the DNA-binding domain of Egr-1 (also known as Zif268) in the free state and in the complex with target DNA. This protein recognizes the target 9-bp DNA sequence via three Cys_2_His_2_-class zinc fingers with high affinity (10). For the Egr-1 DNA-binding domain, the dissociation constant of the specific DNA complexes ranges from 10^−11^ M to 10^−8^ M, depending on ionic strength (11–13). In the brain, Egr-1 is induced by synaptic signals and activates genes for long-term memory formation and consolidation (14,15). In the cardiovascular system, Egr-1 is a stress-inducible transcription factor that activates genes for initiating defense responses against vascular stress and injury (16,17). The Egr-1–DNA interactions were extensively characterized in previous biophysical and biochemical studies (12,13,18–21) and high-resolution crystal structures are available for the Egr-1–DNA complexes (22–24). The investigations at an atomic level are important particularly because Egr-1 (Zif268) has been used as a major scaffold for zinc-finger (ZF) technology for artificial gene editing and regulation (25–27).

**Figure 1.**
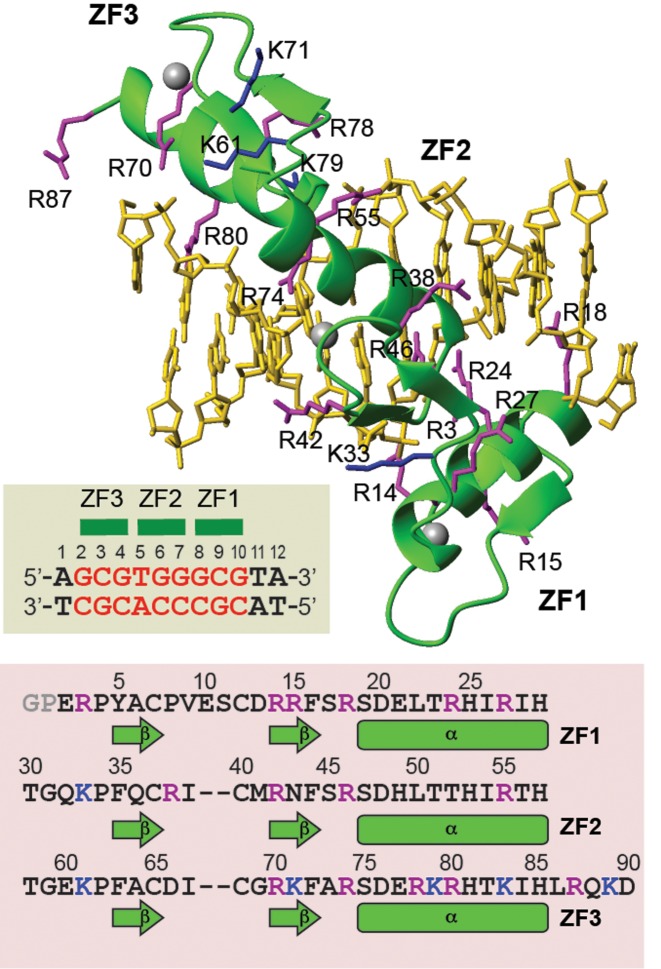
The Egr-1 (Zif268) zinc-finger (ZF)–DNA complex studied in this work. The structure shown is PDB ID: 1AAY (22). The 12-bp DNA duplex contains the target sequence (red) recognized by Egr-1. The ZF domains contain 15 Arg and 6 Lys side chains, which are shown in purple and blue, respectively. The residue numbering schemes are according to Pabo *et al*. (10,22).

In this work, we investigate the internal motions of Lys side-chain NH_3_^+^ and Arg guanidino N_ϵ_-H_ϵ_ moieties in the free and DNA-bound states using NMR spectroscopy and examine changes in mobility of each basic side chain upon Egr-1's binding to the target DNA. The ZF DNA-binding domain of Egr-1 contains 21 basic side chains (15 Arg and 6 Lys residues), 15 of which interact with DNA (Figure 1). The cationic groups exhibit well-isolated NMR signals in ^1^H-^15^N heteronuclear correlation spectra for both the free protein and the complex. Thus, this system provides an opportunity for in-depth investigations on dynamic behavior of each basic side chain in the DNA recognition process. Our NMR data provide comprehensive experimental data on changes in conformational dynamics of basic side chains upon protein–nucleic acid association. In conjunction with NMR, we also use molecular dynamics (MD) simulations to gain deeper insight into the side-chain dynamics and conformational entropy in the protein–DNA association process. Characteristic differences between Arg and Lys side chains in DNA recognition dynamics become evident through these experimental and computational investigations.

## MATERIALS AND METHODS

### Protein and DNA preparation

The Egr-1 ZF protein comprised of three zinc fingers (human Egr-1 residues 335–423) was prepared as described previously. ^15^N or ^13^C/^15^N labeled proteins were expressed in *Escherichia coli* strain BL21 (DE3) cultured in minimal media containing ammonium chloride and glucose as sole nitrogen and carbon sources. The unlabeled 12-bp DNA duplex of dAGCGTGGGCGAT and dATCGCCCACGCT (underline, the Egr-1 recognition site) was chemically synthesized and purified as described (28). NMR samples of the Egr-1–DNA complex were 370-μl solutions containing 0.4 mM protein and 0.6 mM DNA in a buffer of 20 mM potassium succinate (pH 5.8), 20 mM KCl, 0.1 mM ZnCl_2_. Based on the dissociation constant of this complex (12) and the concentrations of Egr-1 and DNA, more than 99.9% of the protein is expected to be in the DNA-bound state under the buffer conditions used. NMR samples of the free protein (0.4 mM) were prepared with the same buffer compositions. Each sample was sealed in a Norell co-axial tube (diameter, 5 mm) in which D_2_O for the NMR lock is separately sealed into the inner stem (diameter, 2 mm) to avoid isotope shifts and broadening of ^15^N resonances due to hydrogen-deuterium exchange (29).

### NMR experiments

NMR experiments were performed with Bruker Avance III NMR spectrometers operated at a ^1^H frequency of 600, 750 or 800 MHz. The 600 and 800-MHz spectrometers were equipped with a cryogenic probe, whereas the 750-MHz spectrometer was equipped with a room-temperature probe. All NMR data were processed with the NMR-Pipe program (30) and analyzed with the NMR-View program (31). For both the free and DNA-bound proteins, backbone ^1^H, ^13^C, and ^15^N resonances were assigned using 3D HNCO, HN(CA)CO, HN(CO)CA, HNCA, CBCA(CO)NH, HNCACB and HBHA(CO)NH spectra (32). Side-chain ^1^H and ^13^C resonances were assigned using 3D HCCH-TOCSY, HCCH-COSY, H(CCO)NH and C(CO)NH spectra (32). These experiments for resonance assignment were performed at 35°C for the complex and at 25°C for the free proteins. Arg side-chain ^15^N_ϵ_ and ^1^H_ϵ_ resonances were assigned using broadband HNCACB and 3D ^15^N-edited NOESY spectra, as described (33). Lys side-chain NH_3_^+^ resonances were assigned using Lys-selective 2D HISQC, H2(C)N, (H2C)N(CC)H-TOCSY, 3D H3NCECD and 3D H3NCG spectra, as described (34). The ^1^H, ^13^C and ^15^N resonance assignment data were deposited to Biological Magnetic Resonance Data Bank: the accession numbers are 26808 for the DNA-bound protein and 26807 for the free protein.

To determine the rotational diffusion parameters, the backbone ^15^N *R*_1_ and *R*_2_ relaxation rates at the ^1^H frequency of 800 MHz were measured for the free protein at 5°C and 25°C and for the complex at 10°C and 25°C.

The ^15^N relaxation experiments for Arg N_ϵ_-H_ϵ_ groups were performed with the pulse sequences for NH groups together with selective ^15^N rSNOB 180° pulses (1.0 ms) (35) in the INEPT schemes. By using ^15^N carrier position set to 81 ppm together with these selective pulses, the Arg N_ϵ_-H_ϵ_ resonances were selectively observed in these ^15^N relaxation experiments. For Arg N_ϵ_-H_ϵ_ groups, ^15^N *R*_1_ and heteronuclear NOE data were recorded at the ^1^H frequencies of 750 and 600 MHz and ^15^N *R*_2_ data were recorded at the ^1^H frequency of 750 MHz. ^15^N *R*_2_ relaxation dispersion experiment for Arg ^15^N_ϵ_ nuclei was performed at the ^1^H frequency of 750 MHz using the CW-CPMG scheme (36) with the CPMG frequencies (*ν_CPMG_*) of 33, 67, 100, 200, 333, 500, 667, 1000, 1333 and 1667 Hz. The Arg relaxation experiments were carried out at 25°C for both the free protein and the complex.

The Lys NH_3_^+^ relaxation experiments were performed as described in our previous publications (18,37–41). For Lys NH_3_^+^ groups, ^15^N *R*_1_ and heteronuclear NOE data were recorded at the ^1^H frequencies of 800 and 600 MHz and ^15^N *R*_2_ data were recorded at the ^1^H frequency of 800 MHz. Lys ^15^N *R*_2_ relaxation dispersion experiment (38) was performed at the ^1^H frequency of 800 MHz with the CPMG frequencies of 33, 67, 100, 200, 333, 500, 667, 1000, 1333 and 1667 Hz. These measurements for the complex were conducted at 10°C under the above-mentioned buffer conditions. The Lys ^15^N relaxation experiments for the free protein were conducted at 5°C and pH 5.0. The lower temperature and pH were necessary to mitigate broadening of the Lys NH_3_^+^ signals due to rapid hydrogen exchange (38,39). On the other hand, the use of 5°C was difficult for the complex because of the poor quality of backbone relaxation data at that temperature. So, we used different temperatures (10 versus 5°C) in the Lys side-chain ^15^N relaxation experiments for the complex and for the free protein. Judging from our previous temperature-dependence study on the internal motions of Lys NH_3_^+^ groups (41), the use of these different temperatures does not significantly interfere with comparative analysis of the NH_3_^+^ order parameters. To detect hydrogen-bond scalar coupling between Lys NH_3_^+^ and DNA phosphate groups, the two-dimensional H3(N)P experiment was performed for the complex at 10°C using a cryogenic QCI-P (^1^H, ^13^C, ^15^N and ^31^P) probe at the ^1^H-frequency of 600 MHz, as described (37).

### ^15^N relaxation data analysis

Rotational diffusion parameters (*D*_||_, *D*_⊥_ and two polar angles for the main principal axis) for the axially symmetric diffusion model (42) were determined from the backbone ^15^N relaxation data using a C program together with GNU Scientific Library, as described (43,44). The effective rotational correlation time *τ_r,eff_* and the anisotropy of the rotational diffusion *r* are given by (2*D*_||_ + 4*D*_⊥_)^−1^ and *D*_||_ / *D*_⊥_, respectively (42). For the free protein, this calculation was performed separately for the three ZF domains because they tumble almost independently in the free state. Using MATLAB software, the order parameters for Arg N_ϵ_-H_ϵ_ groups were calculated from the relaxation data at the two magnetic fields. The ^15^N chemical shift anisotropy parameter (*σ*_||_ - *σ*_⊥_) for arginine side-chain ^15^N_ϵ_ nuclei was set to −114 ppm and the N_ϵ_-H_ϵ_ distance was set to 1.04 Å according to Trbovic *et al*. (45). Four spectral density functions were tested for each Arg N_ϵ_-H_ϵ_ group: two of them were the model-free functions of Lipari and Szabo (Equations. 35 and 43 in Ref. (46)) and the others were the extended model-free functions of Clore *et al*. (Equations 2 and 4 in Ref. (47) multiplied by 2/5). The best model among the four spectral density functions was selected using Akaike's information criterion calculated for each model (48). Using Mathematica software, the order parameters for Lys side-chain NH_3_^+^ groups were calculated from the ^15^N relaxation data at the two magnetic fields, as previously described in detail by Esadze *et al*. (38).

### Molecular dynamics simulations

MD simulations of the Egr-1–DNA complex and the free Egr-1 solvated with TIP3P water molecules were performed using NAMD 2.9 software (49) with CHARMM27 all-atom force fields parameters (50–52), as previously described (18). The 1.6 Å resolution crystal structure of the Egr-1–DNA complex (PDB ID: 1AAY) (22) was used for initial structures. For each system, the macromolecule was solvated in a box of TIP3P water molecules of suitable dimensions: 69.0 × 73.0 × 74.0 Å^3^ (the complex) and 85.8 × 89.3 × 91.3 Å^3^ (free protein). For the free protein, a larger water box was introduced to ensure all possible conformational states are sufficiently solvated, as the inter-domain displacement became increasingly extended in the first 100 ns from the initial compact structure. For zinc-coordinating cysteine residues, the parameters of the deprotonated thiolate moieties were taken from Foloppe *et al*. (53). The parameters for the zinc ions were set based on the hydration free energy parameter set of Merz *et al*. (54). The tautomeric state with protonated N_δ1_ and deprotonated N_ϵ2_ atoms was used for zinc-coordinating histidine residues. The protonation states of other titratable residues were assigned according to their standard protonation states at pH 7.0. The Na^+^ and Cl^−^ ions were randomly added to neutralize the system at the salt concentration of 0.15 M. Particle Mesh Ewald was used to calculate long-range electrostatic interactions, and van der Waals interactions were truncated at 12 Å. All bonds were constrained using the SETTLE algorithm with a time step of 2 fs. Temperature was controlled with Langevin dynamics with a damping coefficient of 5 ps^−1^. The Nosé-Hoover method with a Langevin piston was used to maintain a pressure of 1 atm with an oscillation period of 100 fs and a damping time of 50 fs. After energy minimization, the systems were first heated from 25 K to 298 K with restraints on the C_α_ atom of the protein in the NVT ensemble, and then were switched to the NPT ensemble. The trajectory was saved at an interval of 0.1 ps, and was continued up to 600 ns for the complex and ∼700 ns for the free protein. For the free protein, the last 600 ns were used for analysis.

### Computation of Lys/Arg order parameters and conformational entropies from MD trajectories

Order parameters for Arg N_ϵ_-H_ϵ_ and Lys C_ϵ_-N_ζ_ bond vectors were calculated from the MD trajectories using the auto-correlation function for internal motions (46,55)

(1) \begin{equation*} {C_I}(t)=\langle{P_2}[{\mu({{t_0}+t})\mu(t)}]\rangle, \end{equation*}

where *μ*(*t*_0_)μ(*t*_0_ + *t*) is the projection of a unit vector pointing along a bond vector at time *t*_0_ onto itself at time *t*_0_ + *t*; *P*_2_(*x*) = (3*x*^2^ – 1)/2, is the second Legendre polynomial; and the brackets denote a time average over the trajectory. The trajectory frames were first superimposed onto a reference to remove the effects of overall tumbling. For the free Egr-1 protein, the reference frame was individually defined for each ZF domain because the three ZF domains exhibit virtually independent domain motions (56). For the complex, the reference frame for the auto-correlation function was defined with principal axes of the complex. The time dependence of the autocorrelation for the reorientational motion was analyzed with Clore's extended model-free auto-correlation function (47):

(2) \begin{equation*} {C_I}(t)={S^2}+\left({1-S_f^2}\right){\rm{exp}}({- t/{\tau _f}}) + \left( {S_f^2 - {S^2}} \right){\rm{exp}}({-t/{\tau _i}}) \end{equation*}

where *S_f_*^2^ and *τ_f_* are the amplitude and correlation time due to fast librational motion, *S*^2^ and *τ_i_* are the order parameter and correlation time of the reorientational motion of a bond vector.

Lys and Arg side-chain conformational entropies were calculated from the distributions of the dihedral angles sampled during the simulations (45,57):

(3) \begin{equation*} {S_{conf}} = - R\int P(\vec{\chi }){\rm{ln}}P( {\vec{\chi }} )d\vec{\chi }, \end{equation*}

where *R* is the gas constant; and $P( {\vec{\chi }} )$ is the probability density as a function of the dihedral angles χ_1_, χ_2_, χ_3_ and χ_4_ of each Arg or Lys side chain. The dependence of the entropies on the bin size of the integral mesh was tested and 5° was chosen. The bin size of the integral mesh has an effect on the absolute entropy, but not on the change in conformational entropy upon complex formation.

## RESULTS

We compare the dynamics of basic side chains of the Egr-1 (Zif268) ZF protein in the free state and in the complex with a 12-bp DNA duplex containing the target sequence. In our previous study, we analyzed the dynamics of Lys side chains in the Egr-1–DNA complex (18). In the current study, we conduct the dynamics investigations for the Arg side chains in the free protein and in the complex as well as for the Lys side chains in the free protein. These data allow us to investigate changes in mobility of each basic side chain upon Egr-1's binding to the target DNA. In the following description of the Egr-1 ZF and the DNA duplex, we adopt the residue-numbering schemes shown in Figure 1, as previously defined by Pabo *et al*. (10,22).

### NMR spectra for side-chain cationic groups of the free protein and the complex

**Figure 2.**
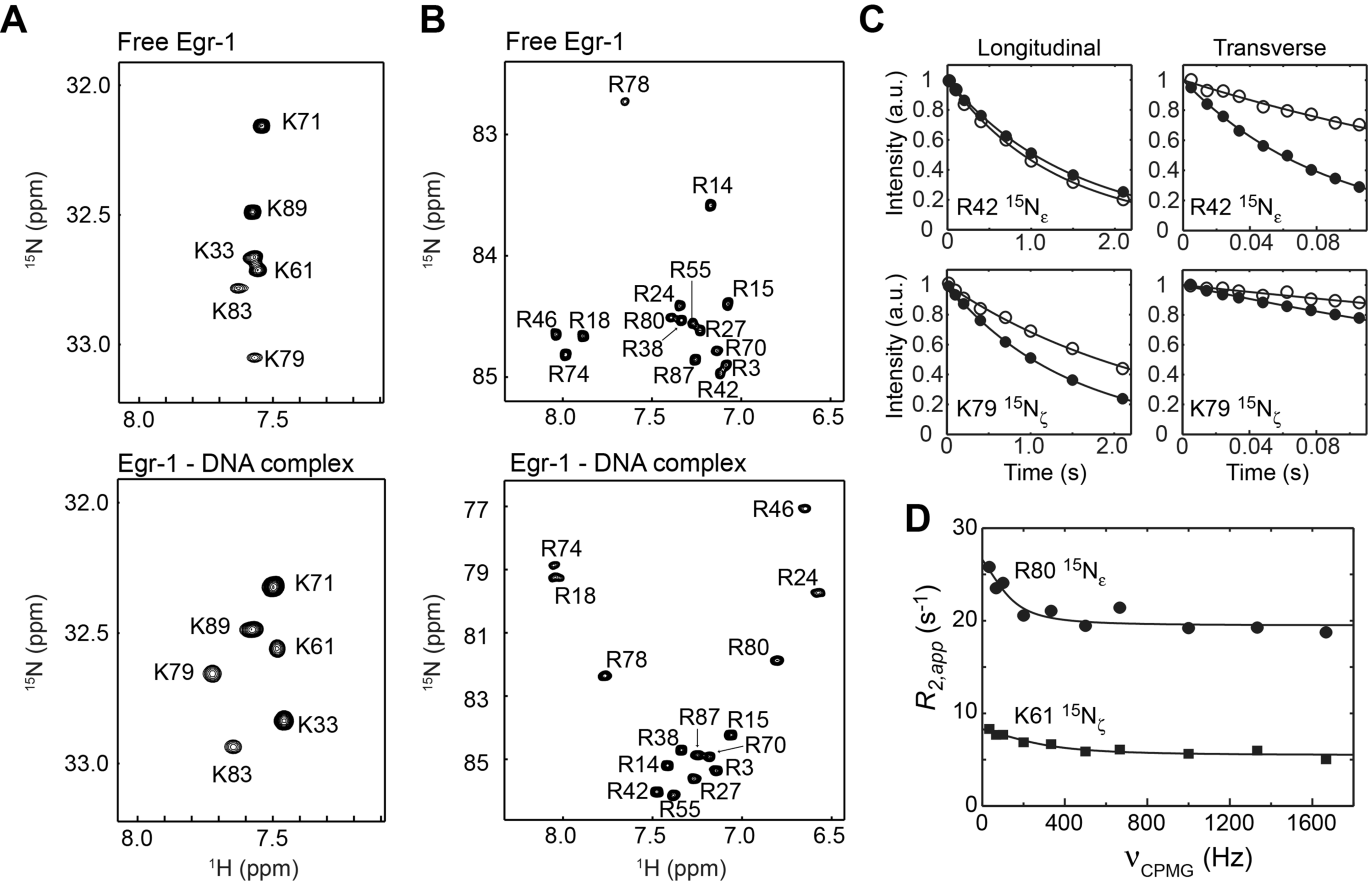
Arg and Lys side-chain heteronuclear ^1^H-^15^N correlation spectra recorded for the free Egr-1 and for the Egr-1 ZF DNA complex. (**A**) Lys NH_3_^+^-selective ^1^H-^15^N heteronuclear in-phase single-quantum coherence (HISQC) spectra (29) recorded for the free and DNA-bound Egr-1 proteins. (**B**) Arg N_ϵ_-H_ϵ_ selective ^1^H-^15^N HISQC spectra. (**C**) Examples of ^15^N longitudinal and transverse relaxation data. Data for the K79 NH_3_^+^ and R42 N_ϵ_-H_ϵ_ groups are shown. Data for the free protein and for the complex are shown with open and closed circles, respectively. (**D**) CPMG *R*_2_ relaxation dispersion data for R80 ^15^N_ϵ_ and K61 ^15^N_ζ_ nuclei of the complex.

As shown in Figure 2A and B, the Lys NH_3_^+^ and Arg N_ϵ_-H_ϵ_ groups of Egr-1 exhibit well-dispersed signals in the ^1^H-^15^N heteronuclear in-phase single quantum coherence (HISQC) spectra for both free and DNA-bound states. For both Arg N_ϵ_-H_ϵ_ and Lys NH_3_^+^ groups, the complex exhibited wider distributions in ^15^N and ^1^H chemical shifts, presumably due to formation of hydrogen bonds and/or ion pairs with DNA. The observation of well-isolated signals for the free and DNA-bound states under the identical (for Arg) or similar (for Lys) conditions allowed us to study the change in dynamics of basic side chains upon DNA-binding.

### ^15^N relaxation data of basic side chains

We measured ^15^N relaxation of Arg N_ϵ_-H_ϵ_ and Lys NH_3_^+^ groups of the free and DNA-bound Egr-1 ZFs. The relaxation experiments for Arg side chains were conducted at the ^1^H frequencies of 750 and 600 MHz; and those for Lys side chains were conducted at 800 and 600 MHz. The relaxation parameters measured for the Arg and Lys side chains are shown in Supplementary Tables S1, 2 and 3 in the Supplementary Data. Figure 2C shows the ^15^N longitudinal and transverse relaxation data and best-fit curves for R42 and K79 side chains in the free protein and in the complex, as typical examples. Despite the smaller molecular size of the free protein, precision in the Lys NH_3_^+^ relaxation measurements for the free protein was slightly worse than that for the complex, presumably due to the faster hydrogen exchange rates. The ^15^N relaxation data for the free protein and the complex were clearly different due to different molecular rotational correlation times as well as due to changes in internal motions of the side chains upon Egr-1 binding to DNA.

### Slow side-chain dynamics detected by CPMG relaxation dispersion experiment

To detect slow dynamics on a μs–ms timescale and analyze their contribution (*R_ex_*) to ^15^N *R*_2_ relaxation rates, we conducted ^15^N *R*_2_ CPMG relaxation dispersion experiments for Arg ^15^N_ϵ_ and Lys ^15^N_ζ_ nuclei. As shown in Figure 2D, the CPMG *R*_2_ relaxation dispersion data for Arg ^15^N_ϵ_ and Lys ^15^N_ζ_ nuclei showed that R80 and K61 undergo slow dynamics in the complex. By applying the two-state fast-exchange model of Loria *et al*. (i.e. Equation 2 in Ref. (58)) to these data, the exchange rates for R80 and K61 were calculated to be (0.8 ± 0.4) × 10^3^ s^−1^ and (1.8 ± 0.5) × 10^3^ s^−1^, respectively. Interestingly, both of these two side chains interact with the Gua2 nucleotide residue. Because terminal base pairs of DNA are know to fray and transiently break the inter-base hydrogen bonds (59), the adjacent Gua2 residue as well as the interacting protein side chains might be influenced by the fraying events. For these residues in the complex, the exchange contribution to *R*_2_ relaxation rates were subtracted from the observed *R*_2_ in the subsequent analysis of the side-chain order parameters. For the other residues, the Arg and Lys relaxation dispersion data did not show any evidence of slow dynamics.

### Order parameters for the Arg and Lys cationic groups

Using the ^15^N relaxation data at two magnetic fields, we determined the order parameters for Arg N_ϵ_-H_ϵ_ and Lys NH_3_^+^ groups of the free Egr-1 protein and the Egr-1–DNA complex. For this analysis, the molecular rotational diffusion parameters were determined from backbone ^15^N relaxation rates *R*_1_ and *R*_2_ together with the 1.6-Å-resolution crystal structure of the complex (PDB ID: 1AAY). The rotational diffusion parameters determined for the free protein and the complex are summarized in Supplementary Table S4 in the Supplementary Data. For the free protein, these parameters were calculated individually for each ZF domain, because the three ZF domains tumble almost independently in the free state, as reported for a similar protein involving three Cys_2_His_2_-class zinc fingers (56). The side-chain ^15^N relaxation data together with the rotational diffusion data were used to determine the order parameters of the Arg N_ϵ_-H_ϵ_ and Lys NH_3_^+^ groups in the free protein and in the protein–DNA complex. Table 1 lists the order parameters determined for the Arg and Lys side chains. In the following three subsections, we describe changes in mobility of these side chains upon Egr-1's binding to DNA. We categorize the basic side chains into three classes: (i) those that electrostatically interact with DNA backbone; (ii) those that interact with DNA bases; and (iii) those outside the protein–DNA interfaces.

**Table 1.** Order parameters determined for Arg N_ϵ_-H_ϵ_ groups^a^ and Lys NH_3_^+^ groups^b^ of Egr-1 in the free and DNA-bound states by NMR

Side chains	*S*^2^ (free protein)	*S*^2^ (complex)
Electrostatically interact with DNA phosphates		
R3	0.215 ± 0.021	0.393 ± 0.009
R14	0.370 ± 0.018	0.292 ± 0.004
R27	0.220 ± 0.010	0.883 ± 0.016
R42	0.386 ± 0.023	0.666 ± 0.010
R55	0.241 ± 0.013	0.899 ± 0.021
R70	0.339 ± 0.027	0.302 ± 0.006
R78	0.399 ± 0.195	0.630 ± 0.025
K33	0.314 ± 0.008	0.378 ± 0.003^c^
K61	0.332 ± 0.011	0.335 ± 0.003^c^
K79	0.276 ± 0.024	0.258 ± 0.005^c^
Interact with DNA bases		
R18	0.295 ± 0.010	0.968 ± 0.020
R24	0.245 ± 0.018	0.968 ± 0.033
R46	0.454 ± 0.022	0.962 ± 0.023
R74	0.306 ± 0.012	0.908 ± 0.039
R80	0.238 ± 0.034	0.897 ± 0.015
Outside the interfaces		
R15	0.462 ± 0.027	0.351 ± 0.004
R38	0.228 ± 0.010	0.108 ± 0.002
R87	0.132 ± 0.024	0.085 ± 0.003
K71	0.279 ± 0.005	0.219 ± 0.003^c^
K83	0.249 ± 0.024	0.322 ± 0.006^c^
K89	0.134 ± 0.003	0.075 ± 0.003^c^

^a^Order parameters were determined from ^15^N relaxation data at ^1^H-frequencies of 750 and 600 MHz. Arg side-chain ^15^N relaxation parameters are reported in Supplementary Table S1.

^b^Order parameters *S*^2^ for the NH_3_^+^ symmetry axis (i.e. the C_ϵ_-N_ζ_ bond vector) were determined from ^15^N relaxation data at ^1^H-frequencies of 800 and 600 MHz. Lys side-chain ^15^N relaxation parameters are reported in Supplementary Table S2.

^c^The Lys NH_3_^+^ order parameters for the complex are from Ref. (18).

### Change in mobility of basic side chains that electrostatically interact with DNA phosphates

**Figure 3.**
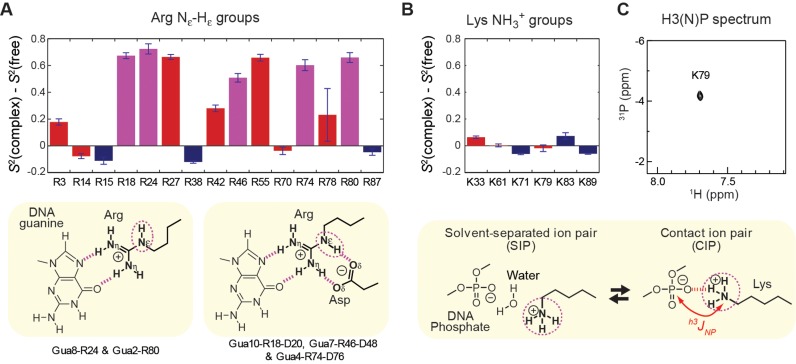
Binding-induced changes in mobility for the Arg N_ϵ_-H_ϵ_ and Lys NH_3_^+^ groups. (**A**) Changes in the Arg N_ϵ_-H_ϵ_ order parameters upon Egr-1's binding to DNA. (**B**) Changes in the Lys NH_3_^+^ order parameters upon Egr-1's binding to DNA. Red, side chains that form intermolecular ion pairs with DNA phosphates; magenta, side chains that form contacts with DNA bases; and blue, side chains located outside the protein–DNA interfaces. (**C**) The H3(N)P spectrum (37) recorded for the Lys NH_3_^+^ groups of the complex, which indicates the presence of the contact ion pair formed by K79 and DNA phosphate. The ^31^P chemical shift is referenced to trimethyl phosphate.

The crystal structures of the Egr-1–DNA complexes (10,22) show short-range electrostatic interactions with the DNA backbone for seven Arg side chains (R3, R14, R27, R42, R55, R70 and R78) and three Lys side chains (K33, K61 and K79). Changes in the order parameters of these cationic groups upon Egr-1's binding to DNA are shown in red in Figure 3A and B. The majority of Arg side chains (i.e. R3, R27, R42, R55 and R78) showed a large increase (by >0.1) in the N_ϵ_-H_ϵ_ order parameter upon the complex formation, indicating that these side-chains become significantly less mobile due to the interactions with DNA. Interestingly, all Lys NH_3_^+^ groups and 2 Arg N_ϵ_-H_ϵ_ groups (R14 and R70) showed no or only marginal changes in their order parameters upon Egr-1's binding to DNA, indicating that the side chains retain high mobility even in the complex. For K79, a ^1^H-^31^P heteronuclear correlation cross peak arising from the hydrogen-bond scalar coupling between the ^31^P and ^15^N nuclei was clearly observed in the H3(N)P spectrum (Figure 3C), indicating the presence of the contact ion pair (CIP) of this side chain and DNA phosphate. Perhaps surprisingly, the K79 NH_3_^+^ group exhibits virtually no change in the order parameter upon DNA-binding, despite the presence of the CIP state in the complex. This high mobility is likely due to the dynamic equilibria between the CIP and solvent-separated ion-pair states (4,18).

### Change in mobility of basic side chains that interact with DNA bases

In the crystal structures of the Egr-1–DNA complexes, 5 Arg side chains (R18, R24, R46, R74 and R80), but no Lys side chain directly interact with DNA bases. Changes in the N_ϵ_-H_ϵ_ order parameters for these Arg side chains are shown in magenta in Figure 3A. The guanidino groups of these Arg side chains form two hydrogen bonds with a guanine base for each (Gua10, Gua8, Gua7, Gua4 and Gua2, respectively; see Figure 3), representing the canonical pattern of guanine recognition by Arg side chain (1,60). Upon formation of the complex with DNA, these Arg side chains exhibited a substantial increase (by >0.5) in the N_ϵ_-H_ϵ_ order parameter *S*^2^, indicating that their mobility is substantially restricted by the interactions with DNA. This strong immobilization is likely due to the two hydrogen bonds at distinct N atoms of the guanidino groups as well as the cation–π interaction (61) with the adjacent base aromatic ring onto which the cationic group stacks.

In addition to the hydrogen bonds with guanine bases, R18, R46 and R74 side chains also form two more hydrogen bonds with an aspartate side chain (i.e. D20, D48 and D76, respectively) (see the scheme in Figure 3). As rigidification of a ligand often increases binding affinity through a decrease in entropic loss upon complex formation (62,63), one might consider that the role of the auxiliary Asp-Arg ion-pair formation at the interface with DNA bases might be to rigidify the Arg side chains to the active conformation in the free protein. However, our NMR data suggest that this is not the case. In fact, the order parameters indicate that R18, R46 and R74 side chains are mobile in the free state (see Table 1).

### Changes in mobility of basic side chains outside the protein–DNA interfaces

In the crystal structures, R15, R38, K71, K83, R87 and K89 are located outside the protein–DNA interfaces and do not directly interact with DNA. Binding-induced changes in the order parameters of these side chains are shown in blue in Figure 3A and B. Egr-1's binding to DNA did not give a significant impact on mobility for a majority of these side chains (i.e. K71, K83, R87 and K89). This is reasonable because they are far from the binding interfaces. Upon binding, R15 and R38 side chains became slightly (but to a statistically significant degree) more mobile. The reason for this mobility is unclear, but might be related to conformational changes of nearby residues. It should be noted that binding-induced enhancement of mobility were previously reported for backbone amide and side-chain methyl groups of other proteins (e.g. Refs. (9,43,64)). The increase in mobility may partially compensate the entropic loss arising from immobilization of many interfacial side chains.

### Comparison with MD simulations

To gain more insight into the side-chain dynamics of Arg and Lys residues, we analyzed dynamic behavior of each basic side chain from the MD simulations. Because we previously obtained a 600-ns MD trajectory for the Egr-1–DNA complex (18), we carried out a corresponding MD simulation of the same length for the free protein in this study. The MD trajectories provide atomic details of side-chain motions and show the contribution from transient interactions, which are not seen in the crystal structures. The direct contacts of these Arg and Lys side chains are summarized in Supplementary Table S5. The mean lifetimes of the direct contacts between the protein and DNA are on the pico to nano-second timescale (see Supplementary Table S6). Arg is much stronger than Lys in participating in direct contacts with the DNA. In addition, based on electrostatic interactions with the phosphate groups of the DNA, the mean lifetimes of the direct contact of Arg appear longer than Lys, which also indicates that these Arg side chains are less dynamic than Lys upon the complex formation.

**Figure 4.**
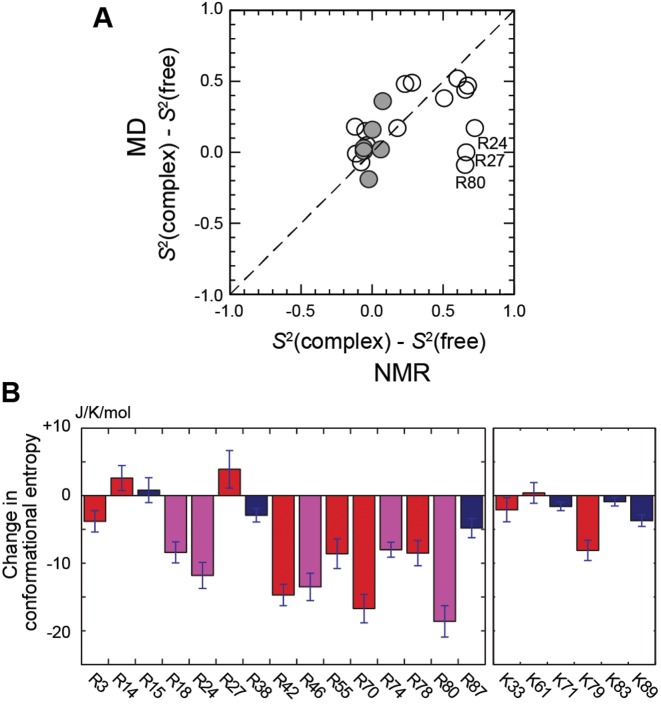
MD trajectory analysis of binding-induced changes in conformational dynamics of the Arg and Lys side chains. (**A**) Comparison of the MD-derived and NMR-derived changes in Arg and Lys side-chain order parameters upon Egr-1's binding to DNA. Data points for Arg and Lys side chains are shown in open and closed circles, respectively. Uncertainties in the NMR-derived changes are shown in Figure 3A and B. (**B**) Binding-induced changes in Arg and Lys side-chain conformational entropies. Each entropy value was calculated from the probability distributions of the dihedral angles *χ*_1_, *χ*_2_, *χ*_3_ and *χ*_4_ in the MD trajectories for the free protein and the Egr-1–DNA complex. Red, side chains that form intermolecular ion pairs with DNA phosphates; magenta, side chains that contact with DNA bases; blue, side chains located outside the protein–DNA interfaces. Error bars represent standard block errors estimated from calculations for independent 50 ns blocks in the 600 ns trajectories for the free protein and the complex.

Using the MD trajectories, we also calculated the order parameters for Arg N_ϵ_-H_ϵ_ and Lys C_ϵ_-N_ζ_ bond vectors in the free and DNA-bound states of Egr-1. Values of the MD order parameters for Arg and Lys side chains are shown in Supplementary Table S7. Figure 4A shows the correlation between binding-induced changes in NMR and MD order parameters. As seen in the results of NMR-based order parameters, the results from the MD trajectory showed a similar trend with relatively large changes in Arg N_ϵ_-H_ϵ_ order parameters and with relatively small changes in Lys C_ϵ_-N_ζ_ order parameters. R24, R27 and R80 gave outliers in the correlation between the experimental data and computation (Figure 4A). For these side chains, although NMR data show a significant increase in N_ϵ_-H_ϵ_ order parameter (i.e. immobilization), the MD simulations show a significantly smaller change. Interestingly, however, even for R24 and R80, the conformational entropy of a whole side chain (not just N_ϵ_-H_ϵ_) showed substantial decreases upon the complex formation (see the following subsection), and in this sense, the computational data were consistent with the experimental observation of the immobilization. The lower MD-derived order parameter of R80 in the complex could be due to the lack of appropriate representation of cation–π interactions in the classical MD force field (as discussed by Schulten *et al*. in Ref. (61)) and/or to fraying of the terminal AT base pair. The intermittent breaking of hydrogen bonds and opening of the terminal base pair have been observed and studied by NMR (59), time-resolved Stokes shifts (65) and computer simulations (66). For R24 and R27, the MD-derived order parameters in the free state were significantly larger than those determined by experiment. This might be related to sampling errors, i.e. lack of convergence of time correlation function due to insufficient conformational/configurational sampling. Long time convergence (100's of ns) of simulations are important for statistical agreement with experimental data for processes on a nanosecond timescale (67). Nonetheless, Figure 4A shows a good correlation between the computational and experimental data for the majority of the basic side chains (18 out of 21), for which the root mean squared difference was 0.19. This supports reliability of the model simulations and justifies further analysis.

### Loss in side-chain conformational entropy of each basic residue upon binding

While the order parameters of Arg N_ϵ_-H_ϵ_ and Lys NH_3_^+^ groups provide information on dynamics of individual side-chain cationic groups, these NMR data do not necessarily reflect mobility in the other parts of Arg and Lys side chains. There are some theoretical models for the relationship between NMR order parameters and conformational entropy (68–70). However, it was proposed that side-chain conformational entropy is not necessarily predictable from NMR order parameters for terminal moieties of long side chains alone because middle parts of the same side chains could remain mobile even if the termini are immobilized (45,71). Since the changes in MD-based order parameters for the cationic moieties were qualitatively consistent with experimental data, we examined Arg and Lys side-chain conformational entropies for the free protein and for the complex using the MD trajectories. To assess the thermodynamic consequences of immobilizing the side-chain cationic groups upon DNA-binding, we calculated side-chain conformational entropy for each basic side chain from the MD trajectories (individual values are reported in Supplementary Table S8 in the Supplementary Data). Figure 4B shows the computed changes in side-chain conformation entropy upon Egr-1's binding to the target DNA. The non-interfacial Arg and Lys side chains showed only marginal changes in conformational entropy. In contrast, many interfacial Arg side chains exhibited significant loss of conformation entropy by ∼8–19 J/mol/K upon protein–DNA association. However, the corresponding entropic loss was smaller for interfacial Lys side chains: K33 and K61 exhibited virtually no loss and K79 exhibited an entropic loss of 8.1 J/mol/K. Thus, these entropic data also illustrate different characteristics of the interfacial Arg and Lys side chains.

## DISCUSSION

### Rigid and dynamic interfaces via basic side chains

This study demonstrates the diverse dynamic properties of the protein–DNA interfaces via basic side chains. The Arg side chains interacting with DNA bases are strongly immobilized and form rigid interfaces. In contrast, despite the strong short-range electrostatic interactions, the majority of the basic side chains interacting with the DNA phosphates are relatively mobile and form dynamic interfaces. In particular, Lys side-chain NH_3_^+^ groups retain high mobility even in the DNA-bound state. Thus, DNA recognition by Egr-1 involves both rigid and dynamic interfaces of the basic side chains.

### High mobility retained by interfacial Lys side chains

It should be entropically favorable that interfacial Lys side chains retain substantial mobility in the DNA-bound state. The retained mobility of Lys side chains could be general in protein–DNA interactions. For example, our previous studies on the HoxD9–DNA and Antp–DNA complexes (18,37,40,41) showed that the interfacial Lys side-chain NH_3_^+^ groups at the molecular interfaces are also highly mobile with *S*^2^_axis_ < 0.6. The small order parameters suggest that binding-induced change in mobility is relatively small for these interfacial Lys NH_3_^+^ groups as well, though the side-chain dynamics of the HoxD9 and Antp proteins in the free state remain to be investigated.

### Different characteristics of Arg and Lys side-chain interactions with DNA

Our data demonstrate the characteristic difference between the interfacial Arg and Lys side chains in the dynamics of DNA recognition. The observed difference can be due to several factors: differences in ability to form hydrogen bonding clusters (1), in charge density, sterics and in desolvation energy (72). For example, pivotal motions through side-chain bond rotations remain possible with a single hydrogen bond, whereas such motions become more difficult with multiple hydrogen bonds. While the multiple hydrogen bonds of an Arg guanidino group with DNA could be favorable in terms of binding enthalpy, the stronger conformational restriction should cause substantial loss in conformational entropy for Arg side chains. In contrast, the Lys side chain possesses only a single charged donor ammonium group, but can adopt various conformations, without substantial loss in side-chain conformational entropy.

This might be partially responsible for different spatial distributions of Arg and Lys side chains at interfaces with DNA. Statistical investigations of the 3D structures of protein-DNA complexes showed that the interactions with DNA minor groove prefer Arg side chains over Lys side chains (72). Roh *et al*. discussed that this preference could be at least partly due to lower desolvation energy for Arg side chains (72). Based on our current data, we speculate that this preference could also relate to the different dynamic properties of Arg and Lys side chains. Insertion in DNA minor groove might diminish the advantage of a Lys side chain in terms of side-chain conformational entropy, because the narrow space in the minor groove would not allow for wide conformational sampling.

## CONCLUDING REMARKS

Our current study delineates the dynamics of the basic side chains in DNA recognition by Egr-1. The Arg side chains interacting with DNA bases are more strongly immobilized and form rigid interfaces. The basic side chains interacting with the DNA phosphates are relatively mobile and form dynamic interfaces. In particular, Lys side-chain NH_3_^+^ groups retain high mobility even in the DNA-bound state. Although the Arg side chains can form a larger number of hydrogen bonds, the strong restriction of their mobility renders substantial loss in side-chain conformational entropy. Our data provide atomic-level information of structural dynamics and thermodynamics of the interfacial Arg and Lys side chains in the DNA-binding event. Although Arg or Lys side-chain dynamics were previously studied for some protein–nucleic acid complexes (18,37,40,41,73–75), the binding-induced changes in the dynamics remained to be delineated. Our current work provides the comprehensive experimental data on changes in Arg and Lys side-chain dynamics upon protein–DNA complex formation. To conclude whether or not the characteristic difference between Arg and Lys side chains are general in molecular recognition of nucleic acids by proteins, we require further investigations of other systems.

## Supplementary Material

SUPPLEMENTARY DATA

## References

[1] Luscombe N.M., Laskowski R.A., Thornton J.M. (2001). Amino acid-base interactions: a three-dimensional analysis of protein-DNA interactions at an atomic level. Nucleic Acids Res..

[2] Nadassy K., Wodak S.J., Janin J. (1999). Structural features of protein-nucleic acid recognition sites. Biochemistry.

[3] Rohs R., Jin X., West S.M., Joshi R., Honig B., Mann R.S. (2010). Origins of specificity in protein-DNA recognition. Annu. Rev. Biochem..

[4] Iwahara J., Esadze A., Zandarashvili L. (2015). Physicochemical properties of ion pairs of biological macromolecules. Biomolecules.

[5] Privalov P.L., Dragan A.I., Crane-Robinson C. (2011). Interpreting protein/DNA interactions: distinguishing specific from non-specific and electrostatic from non-electrostatic components. Nucleic Acids Res..

[6] Record M.T., Ha J.H., Fisher M.A. (1991). Analysis of equilibrium and kinetic measurements to determine thermodynamic origins of stability and specificity and mechanism of formation of site-specific complexes between proteins and helical DNA. Methods Enzymol..

[7] Frederick K.K., Marlow M.S., Valentine K.G., Wand A.J. (2007). Conformational entropy in molecular recognition by proteins. Nature.

[8] Marlow M.S., Dogan J., Frederick K.K., Valentine K.G., Wand A.J. (2010). The role of conformational entropy in molecular recognition by calmodulin. Nat. Chem. Biol..

[9] Tzeng S.R., Kalodimos C.G. (2012). Protein activity regulation by conformational entropy. Nature.

[10] Pavletich N.P., Pabo C.O. (1991). Zinc finger-DNA recognition: crystal structure of a Zif268-DNA complex at 2.1 A. Science.

[11] Elrod-Erickson M., Pabo C.O. (1999). Binding studies with mutants of Zif268. Contribution of individual side chains to binding affinity and specificity in the Zif268 zinc finger-DNA complex. J. Biol. Chem..

[12] Esadze A., Iwahara J. (2014). Stopped-flow fluorescence kinetic study of protein sliding and intersegment transfer in the target DNA search process. J. Mol. Biol..

[13] Hamilton T.B., Borel F., Romaniuk P.J. (1998). Comparison of the DNA binding characteristics of the related zinc finger proteins WT1 and EGR1. Biochemistry.

[14] Bozon B., Davis S., Laroche S. (2003). A requirement for the immediate early gene zif268 in reconsolidation of recognition memory after retrieval. Neuron.

[15] Lee J.L., Everitt B.J., Thomas K.L. (2004). Independent cellular processes for hippocampal memory consolidation and reconsolidation. Science.

[16] Khachigian L.M., Lindner V., Williams A.J., Collins T. (1996). Egr-1-induced endothelial gene expression: a common theme in vascular injury. Science.

[17] Yan S.F., Fujita T., Lu J., Okada K., Shan Zou Y., Mackman N., Pinsky D.J., Stern D.M. (2000). Egr-1, a master switch coordinating upregulation of divergent gene families underlying ischemic stress. Nat. Med..

[18] Chen C.Y., Esadze A., Zandarashvili L., Nguyen D., Pettitt B.M., Iwahara J. (2015). Dynamic Equilibria of Short-Range Electrostatic Interactions at Molecular Interfaces of Protein-DNA Complexes. J. Phys. Chem. Lett..

[19] Esadze A., Kemme C.A., Kolomeisky A.B., Iwahara J. (2014). Positive and negative impacts of nonspecific sites during target location by a sequence-specific DNA-binding protein: origin of the optimal search at physiological ionic strength. Nucleic Acids Res..

[20] Zandarashvili L., Esadze A., Vuzman D., Kemme C.A., Levy Y., Iwahara J. (2015). Balancing between affinity and speed in target DNA search by zinc-finger proteins via modulation of dynamic conformational ensemble. Proc. Natl. Acad. Sci. U.S.A..

[21] Zandarashvili L., Vuzman D., Esadze A., Takayama Y., Sahu D., Levy Y., Iwahara J. (2012). Asymmetrical roles of zinc fingers in dynamic DNA-scanning process by the inducible transcription factor Egr-1. Proc. Natl. Acad. Sci. U.S.A..

[22] Elrod-Erickson M., Rould M.A., Nekludova L., Pabo C.O. (1996). Zif268 protein-DNA complex refined at 1.6 Å: a model system for understanding zinc finger-DNA interactions. Structure.

[23] Hashimoto H., Olanrewaju Y.O., Zheng Y., Wilson G.G., Zhang X., Cheng X. (2014). Wilms tumor protein recognizes 5-carboxylcytosine within a specific DNA sequence. Genes Dev..

[24] Zandarashvili L., White M.A., Esadze A., Iwahara J. (2015). Structural impact of complete CpG methylation within target DNA on specific complex formation of the inducible transcription factor Egr-1. FEBS Lett..

[25] Durai S., Mani M., Kandavelou K., Wu J., Porteus M.H., Chandrasegaran S. (2005). Zinc finger nucleases: custom-designed molecular scissors for genome engineering of plant and mammalian cells. Nucleic Acids Res..

[26] Pabo C.O., Peisach E., Grant R.A. (2001). Design and selection of novel Cys2His2 zinc finger proteins. Annu. Rev. Biochem..

[27] Urnov F.D., Rebar E.J., Holmes M.C., Zhang H.S., Gregory P.D. (2010). Genome editing with engineered zinc finger nucleases. Nat. Rev. Genet..

[28] Takayama Y., Sahu D., Iwahara J. (2010). NMR studies of translocation of the Zif268 protein between its target DNA Sites. Biochemistry.

[29] Iwahara J., Jung Y.S., Clore G.M. (2007). Heteronuclear NMR spectroscopy for lysine NH_3_ groups in proteins: unique effect of water exchange on ^15^N transverse relaxation. J. Am. Chem. Soc..

[30] Delaglio F., Grzesiek S., Vuister G.W., Zhu G., Pfeifer J., Bax A. (1995). NMRPipe—a Multidimensional Spectral Processing System Based on Unix Pipes. J. Biomol. NMR.

[31] Johnson B.A., Blevins R.A. (1994). Nmr View—a computer-program for the visualization and analysis of Nmr data. J. Biomol. NMR.

[32] Clore G.M., Gronenborn A.M. (1998). Determining the structures of large proteins and protein complexes by NMR. Trends Biotechnol..

[33] Iwahara J., Clore G.M. (2006). Sensitivity improvement for correlations involving arginine side-chain Nε/Hε resonances in multi-dimensional NMR experiments using broadband ^15^N 180° pulses. J. Biomol. NMR.

[34] Esadze A., Zandarashvili L., Iwahara J. (2014). Effective strategy to assign ^1^H- ^15^N heteronuclear correlation NMR signals from lysine side-chain NH_3_^+^ groups of proteins at low temperature. J. Biomol. NMR.

[35] Kupče E., Boyd J., Campbell I.D. (1995). Short selective pulses for biochemical applications. J. Magn. Reson. B.

[36] Hansen D.F., Vallurupalli P., Kay L.E. (2008). An improved 15N relaxation dispersion experiment for the measurement of millisecond time-scale dynamics in proteins. J. Phys. Chem. B.

[37] Anderson K.M., Esadze A., Manoharan M., Brüschweiler R., Gorenstein D.G., Iwahara J. (2013). Direct observation of the ion-pair dynamics at a protein-DNA interface by NMR spectroscopy. J. Am. Chem. Soc..

[38] Esadze A., Li D.W., Wang T., Brüschweiler R., Iwahara J. (2011). Dynamics of lysine side-chain amino groups in a protein studied by heteronuclear ^1^H-^15^N NMR spectroscopy. J. Am. Chem. Soc..

[39] Zandarashvili L., Esadze A., Iwahara J. (2013). NMR studies on the dynamics of hydrogen bonds and ion pairs involving lysine side chains of proteins. Adv. Protein Chem. Struct. Biol..

[40] Zandarashvili L., Nguyen D., Anderson K.M., White M.A., Gorenstein D.G., Iwahara J. (2015). Entropic enhancement of protein-DNA affinity by oxygen-to-sulfur substitution in DNA phosphate. Biophys. J..

[41] Zandarashvili L., Iwahara J. (2015). Temperature dependence of internal motions of protein side-chain NH_3_^+^ groups: insight into energy barriers for transient breakage of hydrogen bonds. Biochemistry.

[42] Woessner D.E. (1962). Nuclear spin relaxation in ellipsoids undergoing rotational Brownian motion. J. Chem. Phys..

[43] Iwahara J., Peterson R.D., Clubb R.T. (2005). Compensating increases in protein backbone flexibility occur when the Dead ringer AT-rich interaction domain (ARID) binds DNA: a nitrogen-15 relaxation study. Protein Sci..

[44] Tjandra N., Feller S.E., Pastor R.W., Bax A. (1995). Rotational diffusion anisotropy of human ubiquitin from N-15 NMR relaxation. J. Am. Chem. Soc..

[45] Trbovic N., Cho J.H., Abel R., Friesner R.A., Rance M., Palmer A.G. (2009). Protein side-chain dynamics and residual conformational entropy. J. Am. Chem. Soc..

[46] Lipari G., Szabo A. (1982). Model-free approach to the interpretation of nuclear magnetic-resonance relaxation in macromolecules.1. Theory and range of validity. J. Am. Chem. Soc..

[47] Clore G.M., Szabo A., Bax A., Kay L.E., Driscoll P.C., Gronenborn A.M. (1990). Deviations from the simple 2-parameter model-free approach to the interpretation of N-15 nuclear magnetic-relaxation of proteins. J. Am. Chem. Soc..

[48] d'Auvergne E.J., Gooley P.R. (2003). The use of model selection in the model-free analysis of protein dynamics. J. Biomol. NMR.

[49] Phillips J.C., Braun R., Wang W., Gumbart J., Tajkhorshid E., Villa E., Chipot C., Skeel R.D., Kale L., Schulten K. (2005). Scalable molecular dynamics with NAMD. J. Comput. Chem..

[50] Foloppe N., MacKerell A.D. (2000). All-atom empirical force field for nucleic acids: I. Parameter optimization based on small molecule and condensed phase macromolecular target data. J. Comput. Chem..

[51] MacKerell A.D., Bashford D., Bellott M., Dunbrack R.L., Evanseck J.D., Field M.J., Fischer S., Gao J., Guo H., Ha S. (1998). All-atom empirical potential for molecular modeling and dynamics studies of proteins. J. Phys. Chem. B.

[52] Mackerell A.D., Feig M., Brooks C.L. (2004). Extending the treatment of backbone energetics in protein force fields: limitations of gas-phase quantum mechanics in reproducing protein conformational distributions in molecular dynamics simulations. J. Comput. Chem..

[53] Foloppe N., Sagemark J., Nordstrand K., Berndt K.D., Nilsson L. (2001). Structure, dynamics and electrostatics of the active site of glutaredoxin 3 from Escherichia coli: comparison with functionally related proteins. J. Mol. Biol..

[54] Li P., Roberts B.P., Chakravorty D.K., Merz K.M. (2013). Rational Design of Particle Mesh Ewald Compatible Lennard-Jones Parameters for +2 Metal Cations in Explicit Solvent. J. Chem. Theory Comput..

[55] Case D.A. (2002). Molecular dynamics and NMR spin relaxation in proteins. Acc. Chem. Res..

[56] Brüschweiler R., Liao X., Wright P.E. (1995). Long-range motional restrictions in a multidomain zinc-finger protein from anisotropic tumbling. Science.

[57] Karplus M., Kushick J.N. (1981). Method for estimating the configurational entropy of macromolecules. Macromolecules.

[58] Loria J.P., Rance M., Palmer A.G. (1999). A relaxation-compensated Carr-Purcell-Meiboom-Gill sequence for characterizing chemical exchange by NMR spectroscopy. J. Am. Chem. Soc..

[59] Nonin S., Leroy J.L., Gueron M. (1995). Terminal base pairs of oligodeoxynucleotides: imino proton exchange and fraying. Biochemistry.

[60] Seeman N.C., Rosenberg J.M., Rich A. (1976). Sequence-specific recognition of double helical nucleic acids by proteins. Proc. Natl. Acad. Sci. U.S.A..

[61] Zou X., Ma W., Solov'yov I.A., Chipot C., Schulten K. (2012). Recognition of methylated DNA through methyl-CpG binding domain proteins. Nucleic Acids Res..

[62] Chaires J.B. (2008). Calorimetry and thermodynamics in drug design. Annu. Rev. Biophys..

[63] Velazquez-Campoy A., Todd M.J., Freire E. (2000). HIV-1 protease inhibitors: enthalpic versus entropic optimization of the binding affinity. Biochemistry.

[64] Lee A.L., Kinnear S.A., Wand A.J. (2000). Redistribution and loss of side chain entropy upon formation of a calmodulin-peptide complex. Nat. Struct. Biol..

[65] Andreatta D., Sen S., Perez Lustres J.L., Kovalenko S.A., Ernsting N.P., Murphy C.J., Coleman R.S., Berg M.A. (2006). Ultrafast dynamics in DNA: “fraying" at the end of the helix. J. Am. Chem. Soc..

[66] Zgarbova M., Otyepka M., Sponer J., Lankas F., Jurecka P. (2014). Base pair fraying in molecular dynamics simulations of DNA and RNA. J. Chem. Theory Comput..

[67] Bowman G.R. (2016). Accurately modeling nanosecond protein dynamics requires at least microseconds of simulation. J. Comput. Chem..

[68] Akke M., Brüschweiler R., Palmer A.G. (1993). NMR order parameters and free-energy—an analytical approach and its application to cooperative Ca^2+^ binding by calbindin-D_9k_. J. Am. Chem. Soc..

[69] Li Z.G., Raychaudhuri S., Wand A.J. (1996). Insights into the local residual entropy of proteins provided by NMR relaxation. Protein Sci..

[70] Yang D.W., Kay L.E. (1996). Contributions to conformational entropy arising from bond vector fluctuations measured from NMR-derived order parameters: application to protein folding. J. Mol. Biol..

[71] Li D.W., Brüschweiler R. (2009). A dictionary for protein side-chain entropies from NMR order parameters. J. Am. Chem. Soc..

[72] Rohs R., West S.M., Sosinsky A., Liu P., Mann R.S., Honig B. (2009). The role of DNA shape in protein-DNA recognition. Nature.

[73] Berglund H., Baumann H., Knapp S., Ladenstein R., Härd T. (1995). Flexibility of an arginine side chain at a DNA-protein interface. J. Am. Chem. Soc..

[74] Wilkinson T.A., Botuyan M.V., Kaplan B.E., Rossi J.J., Chen Y. (2000). Arginine side-chain dynamics in the HIV-1 rev-RRE complex. J. Mol. Biol..

[75] Wilkinson T.A., Zhu L., Hu W., Chen Y. (2004). Retention of conformational flexibility in HIV-1 Rev-RNA complexes. Biochemistry.

