# Supplementary material for: Changes in conformational dynamics of basic side chains upon protein–DNA association
Source: Nucleic Acids Res. 2016 Jun 10;44(14):6961–70. doi: 10.1093/nar/gkw531 (PMC5001603; doi:10.1093/nar/gkw531)
Supplement: SUPPLEMENTARY DATA [file supp_44_14_6961__index.html]

Changes in conformational dynamics of basic side chains upon protein–DNA association — Changes in conformational dynamics of basic side chains upon protein–DNA association — SUPPLEMENTARY DATA 

# Changes in conformational dynamics of basic side chains upon protein–DNA association

## SUPPLEMENTARY DATA

- SUPPLEMENTARY DATA
