# Supplementary material for: Changes in conformational dynamics of basic side chains upon protein–DNA association
Source: Nucleic Acids Res. 2016 Jun 10;44(14):6961–70. doi: 10.1093/nar/gkw531 (PMC5001603; doi:10.1093/nar/gkw531)
Supplement: SUPPLEMENTARY DATA [file supp_gkw531_nar-00877-h-2016-File006.pdf]

SUPPORTING INFORMATION

Changes in conformational dynamics of basic side chains upon protein-DNA association

Alexandre Esadze, Chuanying Chen, Levani Zandarashvili, Sourav Roy,
B. Montgomery Pettitt, and Junji Iwahara

Table S1. ^{15}N NMR relaxation data for the Arg side-chain N_ε-H_ε groups of the free Egr-1 protein at 25˚C.

Arg N_ε-H_ε	$^{15}N_\varepsilon$ R_1 (s^{-1}) 600 MHz	$^{15}N_\varepsilon$ R_1 (s^{-1}) 750 MHz	$^{15}N_\varepsilon$ R_2 (s^{-1}) 750 MHz	{^{1}H-}$^{15}N_\varepsilon$ NOE 600 MHz	{^{1}H-}$^{15}N_\varepsilon$ NOE 750 MHz
R3	0.71 ± 0.01	0.62 ± 0.01	2.20 ± 0.13	-0.93 ± 0.03	-0.69 ± 0.05
R14	1.01 ± 0.01	0.85 ± 0.01	2.79 ± 0.11	-0.12 ± 0.02	0.00 ± 0.03
R15	1.11 ± 0.01	0.90 ± 0.01	4.02 ± 0.07	-0.04 ± 0.02	0.06 ± 0.03
R18	0.86 ± 0.01	0.74 ± 0.01	2.65 ± 0.08	-0.52 ± 0.02	-0.37 ± 0.03
R24	0.83 ± 0.01	0.75 ± 0.01	2.70 ± 0.14	-0.67 ± 0.03	-0.36 ± 0.04
R27	0.71 ± 0.01	0.62 ± 0.01	2.32 ± 0.09	-0.89 ± 0.02	-0.66 ± 0.03
R38	0.64 ± 0.01	0.57 ± 0.01	2.45 ± 0.09	-0.83 ± 0.02	-0.64 ± 0.03
R42	0.88 ± 0.01	0.77 ± 0.01	3.53 ± 0.16	-0.27 ± 0.03	-0.18 ± 0.05
R46	0.98 ± 0.01	0.82 ± 0.01	4.07 ± 0.10	-0.07 ± 0.02	0.01 ± 0.03
R55	0.69 ± 0.01	0.59 ± 0.01	2.57 ± 0.10	-0.80 ± 0.02	-0.60 ± 0.03
R70	0.85 ± 0.01	0.77 ± 0.02	3.12 ± 0.23	-0.32 ± 0.03	-0.21 ± 0.05
R74	0.85 ± 0.01	0.72 ± 0.01	3.13 ± 0.07	-0.52 ± 0.02	-0.36 ± 0.03
R78	1.47 ± 0.08	1.12 ± 0.08	3.57 ± 0.66	0.24 ± 0.06	0.32 ± 0.09
R80	0.85 ± 0.01	0.73 ± 0.02	2.17 ± 0.23	-0.58 ± 0.03	-0.35 ± 0.05
R87	0.48 ± 0.01	0.45 ± 0.01	1.67 ± 0.07	-1.66 ± 0.03	-1.31 ± 0.04

Table S2. ^{15}N NMR relaxation data for Lys side-chain NH_3^+ groups of the free Egr-1 protein at 5˚C.

Lys NH_3^+	$^{15}N_\zeta$ R_1 (s^{-1}) 600 MHz	$^{15}N_\zeta$ R_1 (s^{-1}) 800 MHz	$^{15}N_\zeta$ $R_{2,ini}$ (s^{-1})$^{a)}$ 800 MHz	{^{1}H-}$^{15}N_\zeta$ NOE 600 MHz	{^{1}H-}$^{15}N_\zeta$ NOE 800 MHz
K33	0.47 ± 0.01	0.43 ± 0.01	1.19 ± 0.04	-2.88 ± 0.03	-2.78 ± 0.03
K61	0.51 ± 0.01	0.46 ± 0.01	1.31 ± 0.04	-2.88 ± 0.03	-2.81 ± 0.03
K71	0.47 ± 0.01	0.44 ± 0.01	1.14 ± 0.01	-2.69 ± 0.02	-2.53 ± 0.01
K79	0.42 ± 0.01	0.38 ± 0.01	1.08 ± 0.07	-2.63 ± 0.05	-2.64 ± 0.05
K83	0.47 ± 0.01	0.44 ± 0.01	1.07 ± 0.06	-2.87 ± 0.05	-2.64 ± 0.04
K89	0.34 ± 0.01	0.33 ± 0.01	0.66 ± 0.01	-3.15 ± 0.02	-2.73 ± 0.01

a) The initial rate for intrinsically bi-exponential ^{15}N transverse relaxation of NH_3^+ (see Esadze *et al.* [2011] *J Am Chem Soc* 133, 909-13).

Corresponding ^{15}N relaxation data for the Lys NH_3^+ groups of the Egr-1–DNA complex at 10˚C were reported in our previous paper: Chen *et al.* [2015] *J Phys Chem Lett* 6, 2733-7.

Table S3. ^{15}N NMR relaxation data for the Arg side-chain N_ε-H_ε groups of the Egr-1–DNA complex at 25˚C.

Arg N_ε-H_ε	$^{15}N_\varepsilon$ R_1 (s^{-1}) 600 MHz	$^{15}N_\varepsilon$ R_1 (s^{-1}) 750 MHz	$^{15}N_\varepsilon$ R_2 (s^{-1}) 750 MHz	{^{1}H-}$^{15}N_\varepsilon$ NOE 600 MHz	{^{1}H-}$^{15}N_\varepsilon$ NOE 750 MHz
R3	0.79 ± 0.01	0.66 ± 0.01	7.34 ± 0.11	-0.21 ± 0.02	-0.02 ± 0.02
R14	0.93 ± 0.01	0.79 ± 0.01	5.96 ± 0.06	-0.21 ± 0.01	-0.04 ± 0.02
R15	0.88 ± 0.01	0.69 ± 0.01	6.79 ± 0.07	0.05 ± 0.01	0.09 ± 0.02
R18	0.82 ± 0.05	0.51 ± 0.03	20.3 ± 0.94	0.65 ± 0.05	0.68 ± 0.10
R24	0.79 ± 0.03	0.58 ± 0.03	15.6 ± 1.2	0.69 ± 0.07	0.76 ± 0.12
R27	0.70 ± 0.02	0.51 ± 0.01	15.8 ± 0.5	0.68 ± 0.04	0.65 ± 0.06
R38	0.53 ± 0.01	0.47 ± 0.01	2.45 ± 0.04	-1.01 ± 0.01	-0.73 ± 0.02
R42	0.88 ± 0.01	0.67 ± 0.01	11.7 ± 0.14	0.26 ± 0.02	0.28 ± 0.03
R46	0.88 ± 0.05	0.59 ± 0.01	25.9 ± 2.4	0.68 ± 0.08	0.62 ± 0.13
R55	0.72 ± 0.02	0.53 ± 0.02	18.4 ± 0.5	0.49 ± 0.04	0.43 ± 0.06
R70	0.82 ± 0.01	0.71 ± 0.01	5.96 ± 0.10	-0.28 ± 0.01	-0.13 ± 0.02
R74	0.80 ± 0.03	0.57 ± 0.07	29.1 ± 4.3	0.65 ± 0.08	0.40 ± 0.15
R78	0.91 ± 0.02	0.67 ± 0.02	11.2 ± 0.4	0.39 ± 0.03	0.52 ± 0.05
R80	0.83 ± 0.02	0.59 ± 0.02	17.7 ± 0.6	0.41 ± 0.05	0.49 ± 0.08
R87	0.51 ± 0.01	0.47 ± 0.01	2.05 ± 0.06	-1.38 ± 0.02	-1.08 ± 0.03

Table S4. Molecular rotational diffusion parameters for the free Egr-1 protein and the Egr-1–DNA complex.[a)]

	$\tau_{r,eff}$ (ns) [b)]	$D_{\|} / D_{\perp}$ [b)]
Free Egr-1 at 5˚C [c)]		
ZF1	8.23 ± 0.02	1.69 ± 0.02
ZF2	10.51 ± 0.05	1.95 ± 0.02
ZF3	8.42 ± 0.02	2.03 ± 0.02
Free Egr-1 at 25˚C [c)]		
ZF1	4.72 ± 0.03	1.53 ± 0.03
ZF2	6.10 ± 0.04	1.68 ± 0.03
ZF3	4.66 ± 0.02	2.34 ± 0.02
Complex at 25˚C		
ZF1-ZF2-ZF3	13.91 ± 0.05	1.59 ± 0.02

a) Determined from backbone ^{15}N R_1 and R_2 relaxation rates for the secondary-structure regions.
b) Parameters for the axially symmetric rotational diffusion model. The effective molecular rotational correlation time $\tau_{r,eff}$ and the anisotropy r are given by $(2 D_{\|} + 4 D_{\perp})^{-1}$.
c) Because the three zinc-finger (ZF) domains tumble almost independently in the free state, the fitting calculation was performed individually for each domain. The larger value $\tau_{r,eff}$ for ZF2 is likely due to restriction via two domains, as discussed in Brüschweiler *et al.* (1995) *Science* 268, 886-9.

Table S5. Frequencies of direct contacts [a)] for the Arg guanidino and Lys amino groups observed in 600-ns MD trajectories for the free Egr-1 protein and the Egr-1-DNA complex.

a.a.	Free protein	Complex	a.a.	Free protein	Complex
R3	NH2 - E2: 0.47<br>NE - E2: 0.08	NH2 - E2: 0.14<br>NE - S17: 0.17<br>NH1 - Gua8: 0.38<br>NH1 - Cyt9: 0.07<br>NH2 - Gua8: 0.51	R14	--	NH1 - Gua7: 0.75<br>NH2 - Gua7: 0.08
R15	NH1 - E2: 0.13<br>NH1 - D13: 0.32<br>NH2 - E2: 0.15<br>NH2 - D13: 0.27<br>NE - E2: 0.05	NH1 - E2: 0.20<br>NH1 - D13: 0.15<br>NH2 - E2: 0.24<br>NH2 - D13: 0.12	R18	NH2 - D20: 0.87<br>NH2 - E21: 0.11<br>NE - D20: 0.35	NH2 - D20: 1.06<br>NE - D20: 0.48<br>NH1 - N7,Gua10: 0.87<br>NH2 - O6,Gua10: 0.32
R24	NH1 - D20: 0.48<br>NH2 - D20: 0.57<br>NH2 - E21: 0.78<br>NE - E21: 0.20	NH2 - E21: 0.42<br>NH1 - O6,Gua8: 0.61<br>NH2 - N7,Gua8: 0.83	R27	NH1 - P34: 0.43	NH1 - Ade52: 0.18<br>NH2 - Ade52: 0.68
R38	--	--	R42	--	NH1 - Gua4: 0.73<br>NH2 - Gua4: 0.58
R46	NH2 - D48: 0.96<br>NE - D48: 0.43	NH2 - D48: 1.01<br>NE - D48: 0.45<br>NH1 - N7,Gua7: 0.85<br>NH2 - O6,Gua7: 0.55	R55	NH2 - E60: 0.17<br>NE - E60: 0.15	NE - E60: 0.20<br>NH1 - Cyt55: 0.97<br>NH2 - Cyt55: 0.77
R70	--	NH1 - Gua2: 0.42<br>NH2 - Gua2: 0.56	R74	NH2 - D48: 0.08<br>NH2 - D76: 0.67<br>NH2 - E77: 0.10<br>NE - D76: 0.30	NH2 - D76: 1.01<br>NE - D76: 0.59<br>NH1 - N7,Gua4: 0.82<br>NH2 - O6,Gua4: 0.41<br>NH2 - N7,Gua4: 0.11
R78	NH1 - D66: 0.25<br>NH1 - T82: 0.37<br>NH2 - D66: 0.60<br>NH2 - A64: 0.15<br>NE - A64: 0.14	NH1 - T82: 0.12<br>NH2 - S75: 0.45<br>NH1 - Cyt57: 0.29<br>NH2 - Cyt57: 0.82	R80	NH1 - D76: 0.19<br>NH1 - E77: 0.14<br>NH2 - D76: 0.34<br>NH2 - E77: 0.92<br>NE - E77: 0.26	NH2 - E77: 1.08<br>NH1 - O6,Gua2: 0.09<br>NH2 - Gua2: 0.78<br>NH1 - N7,Gua2: 0.11
R87	--	--			
K33	--	--	K61	--	--
K71	E60: 0.35	--	K79	--	Cyt57: 0.34<br>Ade58: 0.19
K83	--	Ade58: 0.51	K89	--	--

[a)] Either a strong salt bridge (SB) or a hydrogen bond (HB). The geometric criteria for a HB between donor (D) and acceptor (A) atoms are 1) the A…H distance < 2.3 Å; 2) the A…D distance < 3.2 Å; and 3) the A…H–D angle being between 130° and 180°. A SB contact is defined as a state with the distance between two charged atoms being less than 2.8 Å. The frequency of each contact is defined as the sum of the occupancies of the equivalent contacts (e.g. those between Arg NH1 and ASP OD1/OD2 atoms), and therefore can exceed 1.0.

Table S6. Mean lifetimes of the direct contacts with DNA for the Arg guanidino and Lys amino groups in the Egr-1-DNA complex (see Table S5).

	R3	R14	R27	R42	R55	R70	R80
τ (ps)[a]	75	70	470	64	249	36	43
	R18	R24	R46	R74	K33	K79	K83
τ (ps)[a]	2	2	2	2	7	20	19

[a.] Mean lifetimes of the contact states. These were estimated by a survival correlation function:

$$S(t) = \langle h(t_0) H(t_0 + t) \rangle / \langle h(t_0) \rangle \quad (1)$$

where $h(t_0)$ is 1 if a contact state is formed at a time origin t_0 , and 0 otherwise. $H(t_0+t)$ is 1 if a contact state remains intact during the period of time t. $S(t)$ provides a strict definition of the lifetime of a contact state, and it is "history – dependent". $S(t)$ was best described by a double exponential model:

$$S(t) = (1 - W)\exp(-t/\tau_{ip-f}) + W\exp(-t/\tau_{ip-s}), \quad (2)$$

where τ_{ip-f} and τ_{ip-s} are the fast and slow correlation times of an ion-pair state, respectively, and W gives the weight of the slow relaxation. It should be noted that the value of the correlation time can be sensitive to the sampling frequency. A long interval between sampled configurations will lead to missing events where a contact is broken for a short time and reform subsequently. In the current work, we sampled every 0.1 ps.

Table S7. Computational order parameters for Arg side-chain N_ε-H_ε and Lys side-chain C_ε-N_ζ bond vectors calculated from the MD trajectories for the for the free Egr-1 and the Egr-1–DNA complex.

Bonds	S^2(free) [a)]	S^2 (complex) [b)]
Arg N_ε-H_ε		
R3	0.24	0.33
R14	0.23	0.15
R15	0.09	0.13
R18	0.42	0.91
R24	0.67	0.78
R27	0.65	0.60
R38	0.07	0.24
R42	0.26	0.69
R46	0.63	0.89
R55	0.55	0.88
R70	0.19	0.35
R74	0.38	0.89
R78	0.37	0.83
R80	0.30	0.36
R87	0.08	0.23
Lys C_ε-N_ζ		
K33	0.07	0.09
K61	0.05	0.21
K71	0.04	0.05
K79	0.39	0.20
K83	0.09	0.45
K89	0.00	0.03

[a)] Calculated from a 600-ns MD trajectory for the free Egr-1 protein. The molecular frame was defined individually for each zinc-finger domain to account for independent domain motions.
[b)] Calculated from a 600-ns MD trajectory for the Egr-1–DNA complex. The molecular frame was defined for the entire complex.

Table S8. Side-chain conformational entropy calculated for Arg and Lys residues from the MD trajectories for the free Egr-1 and the Egr-1–DNA complex.

Side chains	S_{cof} (free) [J/K/mol][a)]	S_{cof} (complex)[J/K/mol][a)]
Arg		
R3	143.7 ± 0.9	139.9 ± 1.3
R14	140.6 ± 1.5	143.2 ± 1.1
R15	145.6 ± 0.9	146.4 ± 1.6
R18	152.2 ± 0.4	143.8 ± 1.5
R24	134.7 ± 1.2	122.9 ± 1.5
R27	131.9 ± 1.2	135.8 ± 2.5
R38	147.6 ± 0.6	144.7 ± 0.8
R42	140.4 ± 0.9	125.7 ± 1.3
R46	143.8 ± 1.1	130.3 ± 1.7
R55	126.5 ± 1.1	117.9 ± 1.9
R70	142.1 ± 1.1	125.4 ± 1.8
R74	151.0 ± 0.5	143.0 ± 1.0
R78	132.4 ± 1.1	123.9 ± 1.5
R80	137.1 ± 1.0	118.5 ± 2.1
R87	146.9 ± 0.3	142.1 ± 1.4
Lys		
K33	140.9 ± 0.8	138.8 ± 1.6
K61	137.3 ± 0.6	137.7 ± 1.4
K71	154.8 ± 0.2	153.2 ± 0.6
K79	142.4 ± 0.2	134.3 ± 1.5
K83	150.9 ± 0.2	150.0 ± 0.6
K89	151.9 ± 0.5	148.2 ± 0.7

[a)] Uncertainties correspond to standard errors estimated from independent 50 - ns blocks of MD trajectories.
